# Dual-functional DHBA/Zn-hybrid electrospun scaffolds for simultaneous nerve repair and bone regeneration

**DOI:** 10.1093/rb/rbag062

**Published:** 2026-03-21

**Authors:** Yiding Shen, Yilong Dong, Peng Hua, Hongyu Luo, Xinkun Shen, Kai Fang, Lingzhuo Hou, Yeyi Zheng, Gaowen Li, Litai Jin, Yongping Yuan, Pingping Ma

**Affiliations:** Ningbo Key Laboratory of Skin Science, Ningbo College of Health Sciences, Ningbo 315000, China; Department of Stomatology, Shaoxing Hospital of Traditional Medicine, Shaoxing 312000, China; The Third Affiliated Hospital of Wenzhou Medical University (Ruian People’s Hospital), Wenzhou 325016, China; Ningbo Key Laboratory of Skin Science, Ningbo College of Health Sciences, Ningbo 315000, China; The Third Affiliated Hospital of Wenzhou Medical University (Ruian People’s Hospital), Wenzhou 325016, China; Ningbo Key Laboratory of Skin Science, Ningbo College of Health Sciences, Ningbo 315000, China; The Third Affiliated Hospital of Wenzhou Medical University (Ruian People’s Hospital), Wenzhou 325016, China; Department of Stomatology, Shaoxing Hospital of Traditional Medicine, Shaoxing 312000, China; Ningbo Key Laboratory of Skin Science, Ningbo College of Health Sciences, Ningbo 315000, China; Ningbo Key Laboratory of Skin Science, Ningbo College of Health Sciences, Ningbo 315000, China; Ningbo Key Laboratory of Skin Science, Ningbo College of Health Sciences, Ningbo 315000, China; Ningbo Key Laboratory of Skin Science, Ningbo College of Health Sciences, Ningbo 315000, China; Ningbo Key Laboratory of Skin Science, Ningbo College of Health Sciences, Ningbo 315000, China; Ningbo Key Laboratory of Skin Science, Ningbo College of Health Sciences, Ningbo 315000, China

**Keywords:** electrospinning scaffold, metal organic framework, neurogenesis, osteogenesis

## Abstract

Oral and maxillofacial surgeries or trauma (e.g. impacted tooth extraction, jaw fracture or tumor resection) often lead to concurrent peripheral nerve injury and bone defects, while current collagen/gelatin sponges offer limited therapeutic effects. To address this challenge, we developed innovative electrospun scaffolds (MOF2, MOF4 and MOF6) by *in situ* synthesis of 3,5-dihydroxybenzoic acid/zinc (DHBA/Zn-MOF) hybrids within a gelatin/polycaprolactone matrix. *In vitro*, Schwann cells treated with material extracts exhibited enhanced migration, regulated myelin-associated genes (*Ngf*/*Pmp22* upregulated, *Ncam* downregulated) and increased NGF protein expression via the PI3K pathway. Co-cultured PC12 cells showed increased neurite outgrowth, confirming neural repair potential. Osteoblasts exposed to material extracts showed elevated alkaline phosphatase activity, enhanced mineralization and upregulated osteogenic genes (*Runx2*, *Alp* and *Opg*), verifying osteogenic capacity. *In vivo*, MOF6 scaffolds achieved superior motor function recovery in a rat sciatic nerve crush model (evidenced by increased compound muscle action potentials and reduced gastrocnemius muscle atrophy) and promoted trabecular bone formation in a rat skull defect model (validated by micro-CT and histological analyses). These findings underscore the dual-functional capability of DHBA/Zn-hybrid scaffolds to simultaneously promote nerve repair and bone regeneration, offering a promising therapeutic approach for complex neuro-bone composite injuries in clinical practice.

## Graphical Abstract

**Graphical Abstract:**
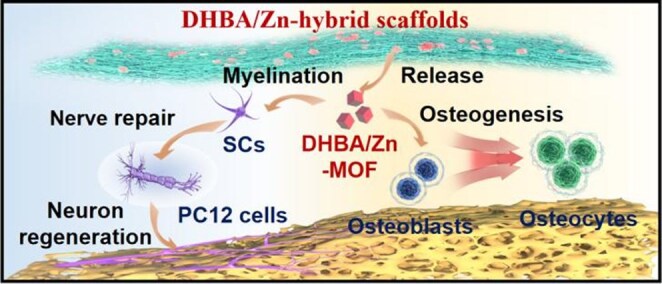

## Introduction

Oral and maxillofacial surgeries or traumatic injuries often involve concurrent damage to peripheral nerves and/or bone tissues, due to the anatomical proximity of neural and skeletal structures in this region [1, 2]. For instance, procedures such as impacted tooth extraction, jaw fracture reduction or oral tumor resection may inevitably cause nerve injury (e.g. inferior alveolar nerve impairment) and bone defects, as substantial tissue manipulation or bone removal is often required [3–5]. Studies have reported that in up to 46.7% of cases involving surgeries for impacted mandibular third molars, the surgical site directly contacts the nerve canal, with another 28.7% showing close adjacency [5, 6]. After extraction, the incidence of inferior alveolar nerve sensory impairment is approximately 6% [7], and 0.28% of patients may suffer permanent damage [8], leading to symptoms such as numbness, drooling or lip biting that significantly affect quality of life. Mecobalamin facilitates neuronal conduction through methylation, while corticosteroids and nonsteroidal anti-inflammatory drugs alleviate perineural edema and inflammation. Analgesics and antidepressants are employed to mitigate neuropathic pain. However, systemic administration of these drugs carries risks of adverse effects, including gastrointestinal ulcers, allergic reactions and renal dysfunction [9, 10]. Localized drug delivery systems offer multiple advantages, including targeted action at the injury site, reduced systemic toxicity, lower dosages and prolonged therapeutic effects [11, 12]. Consequently, sustained local drug delivery has emerged as an optimal strategy to synergistically promote nerve repair and bone regeneration in post-extraction bone defects.

Beyond nerve injury, bone resorption is another common complication following oral and maxillofacial surgeries, primarily due to the loss of mechanical support and functional stimulation from the original tissue [13, 14]. The surgical process can also damage the periosteum and surrounding soft tissues, triggering retrograde axonal degeneration in peripheral nerve endings [15, 16]. Sensory neurons play a key role in maintaining bone homeostasis by releasing neuropeptides. When these neurons degrade after nerve injury, neuropeptide secretion decreases, disrupting bone metabolic balance [17]. Current clinical treatments [e.g. gelatin (Gel) sponges] primarily function as hemostatic agents with limited efficacy in nerve repair or bone defect healing. These limitations underscore the urgent need for advanced biodegradable materials. Such materials should locally release active substances to simultaneously promote neuroregeneration and osteogenesis, addressing the multiple challenges of recovery from neuro-bone composite injuries.

The ‘drug-device’ combination strategy represents a promising approach for localized and sustained drug delivery, addressing critical challenges in clinical practice [18]. Metal–organic frameworks (MOFs), with their highly ordered three-dimensional structures and versatile functions, have garnered increasing attention as biomaterials [19, 20]. Zinc ions (Zn^2+^), known for their exceptional osteogenic properties, have been incorporated into MOF materials to enhance bone regeneration. For instance, Liu *et al.* developed a multifunctional hydrogel incorporating ZIF-8 nanoparticles, which significantly upregulated the expression of alkaline phosphatase (ALP), collagen and osteocalcin, thereby promoting the osteogenic differentiation of bone marrow mesenchymal stem cells [21]. Furthermore, our previous research has successfully developed composite MOF particles doped with sodium alendronate, synthesized *in situ* on electrospun polycaprolactone (PCL)/Gel fibers. These materials demonstrated sustained drug release and robust anti-osteoporotic and osteogenic activities [22]. Despite these advances, current MOF materials have limitations, including rapid degradation rates and suboptimal cellular compatibility, necessitating further optimization of their bioactivity. Notably, MOF materials designed specifically for nerve injury repair remain largely unexplored. Emerging studies on bioactive compounds from apples have identified 3,5-dihydroxybenzoic acid (DHBA) as a potent agonist of the hydroxycarboxylic acid receptor 1, with a receptor affinity surpassing that of lactate [23]. DHBA demonstrates significant neurogenic potential by promoting neural precursor cell proliferation, differentiation and survival [24]. Furthermore, its phenolic hydroxyl and carboxyl groups facilitate self-assembly with metal ions, enabling the synthesis of MOFs [25, 26]. These properties make DHBA an excellent candidate for developing novel DHBA/Zn-MOF hybrid materials. We constructed this material by coordinating DHBA with osteogenically active Zn^2+^. It integrates superior biocompatibility with synergistic neuroregenerative and osteogenic functions, aiming to achieve dual-functional tissue regeneration.

**Figure 1:**
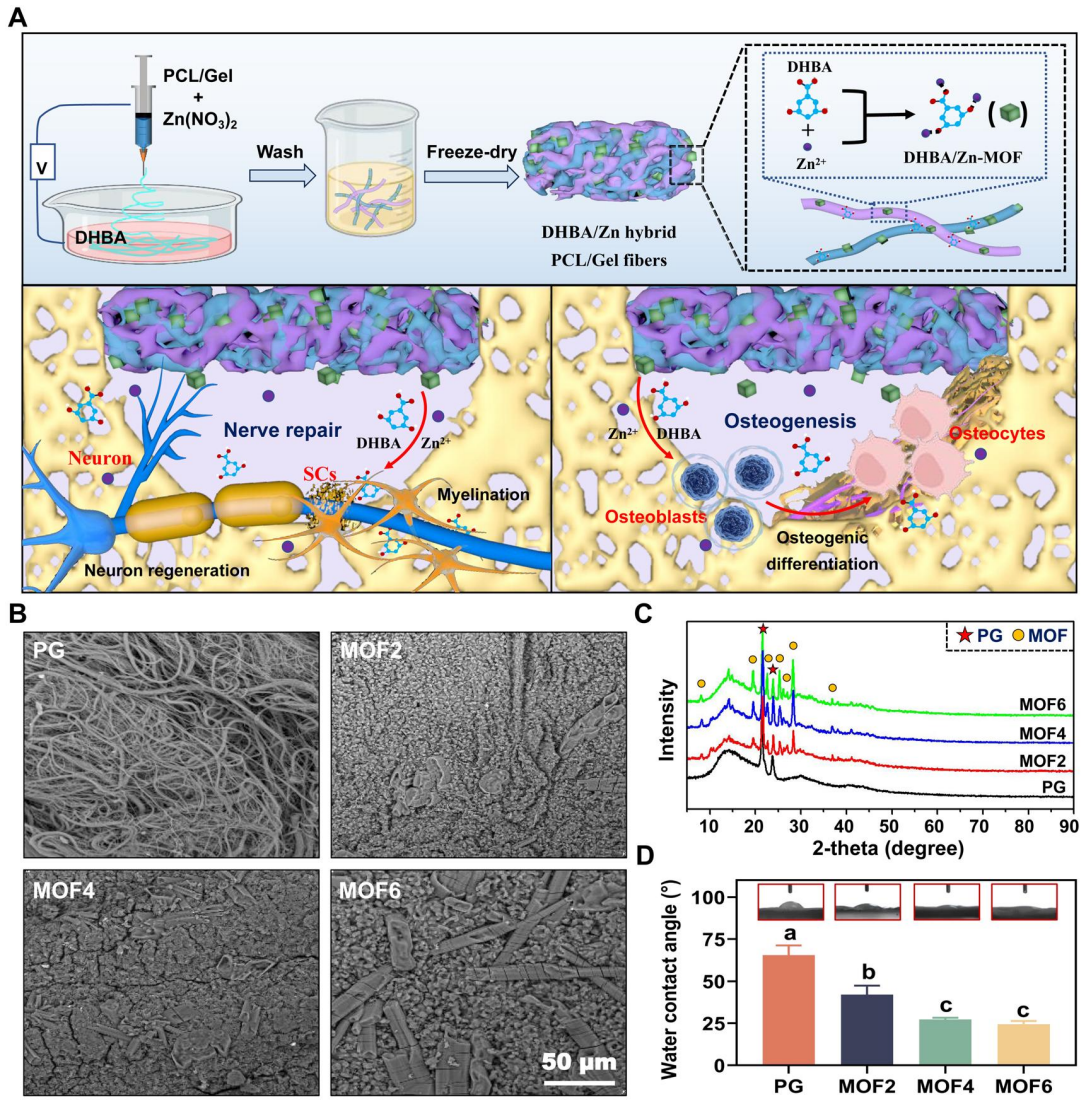
(**A**) Schematic illustration of the fabrication process for DHBA/Zn-hybrid Gel/PCL electrospun scaffolds and its functions in promoting the regeneration of damaged nerves and bone tissue, (**B**) representative scanning electron microscopy (SEM) images, (**C**) X-ray diffraction (XRD) patterns and (**D**) water contact angle measurements for each group of samples. Groups labeled with identical letters (**a**, **b**) indicate no significant differences, while distinct letters signify statistically significant differences (*P *< 0.05).

Electrospinning technology, widely acknowledged for its capacity to fabricate micro- and nano- fibers, has evolved into a preeminent strategy for constructing biodegradable polymeric scaffolds [27, 28]. In contrast to conventional Gel or collagen sponges, which are constrained by limited functional versatility and short degradation cycles, electrospun materials can be tailored through structural optimization and drug incorporation to meet diverse clinical requirements. Based on this technological advantage, we hypothesize that DHBA and Zn^2+^ can undergo *in situ* to form DHBA/Zn-MOF particles within PCL/Gel electrospun scaffolds. It is anticipated that these hybrid scaffolds will exert synergistic effects in facilitating nerve repair and osteogenesis, thereby addressing the multifaceted challenges associated with post-extraction tissue recovery (Figure 1A).

## Materials and methods

### Preparation of electrospun fillers

For the preparation of electrospinning solutions, PCL was dissolved in 2,2,2-trifluoroethyl alcohol (TFE) to prepare a 10 wt% solution, while Gel was dissolved in deionized water to form a 20 wt% aqueous solution. Leveraging the favorable solubility of both PCL and Gel in TFE, as well as the complete miscibility of TFE with water in any proportions, the volume ratio of PCL/TFE solution to Gel/water dispersion was optimized to 7:3 (v/v) based on a previous study [22]. This optimized ratio effectively inhibits phase separation and Gel denaturation [22]. It should be emphasized that Gel, as a hydrolytic derivative of collagen, inherently lacks an intact tertiary structure in aqueous solutions. Upon exposure to TFE, Gel undergoes only reversible conformational transitions (e.g. interconversion between random coils and partial α-helices) rather than irreversible denaturation, thus eliminating the risk of protein structural impairment. To further ensure solution homogeneity (a prerequisite for mitigating phase separation during electrospinning), the mixed solution was stirred at 300 rpm for 12 h at 25°C to achieve uniform dispersion of PCL and Gel molecules. Subsequently, zinc nitrate [Zn(NO_3_)_2_] was added to the homogeneous mixture at final concentrations of 2, 4 and 6 mg/mL, followed by an additional 2 h of stirring to ensure the uniform dispersion of Zn^2+^.

A one-step wet electrospinning process was designed to synergistically achieve Gel/PCL fiber electrospinning and *in situ* DHBA/Zn-MOF coordination synthesis in a single step (Figure 1A), eliminating the need for post-electrospinning modification steps. Electrospinning was performed under optimized parameters: applied voltage of 20 kV, needle-to-collector distance of 10 cm and ambient temperature of 25–35°C. A critical design feature of this strategy is that electrospun fibers were directly deposited into 40 mg/mL DHBA ethanol solution (Aladdin Co. Ltd., China), which simultaneously served as the reaction medium for MOF synthesis. Specifically, upon fiber formation and deposition, the phenolic hydroxyl and carboxyl groups of DHBA in the collection solution immediately chelated with pre-dispersed Zn^2+^ in the fibers, driving *in situ* nucleation and growth of DHBA/Zn-MOF particles on the fiber surfaces via stable metal–ligand coordination bonds. This synchronous integration of electrospinning and MOF synthesis embodies the essence of the ‘one-step’ process by obviating additional post-treatment steps. After the one-step process, the fibers with *in situ* grown DHBA/Zn-MOF particles were molded, compressed and freeze-dried to obtain target fillers. Based on the initial concentration of Zn(NO_3_)_2_ (0, 2, 4, 6 mg/mL), the samples were designed as samples were designated as PG (control, no DHBA/Zn-MOF), MOF2 [2 mg/mL Zn(NO_3_)_2_], MOF4 [4 mg/mL Zn(NO_3_)_2_] and MOF6 [6 mg/mL Zn(NO_3_)_2_], respectively. Finally, scanning electron microscopy (SEM, Zeiss AURIGA FIB, Germany) was performed at 50 kV to analyze the surface morphology. The crystal structure was determined by an X-ray diffractometer (XRD, Rigaku Ltd., Japan). The wettability of the scaffolds was measured using a contact angle measuring instrument (DSA30, Kruss, Germany).

### Release of 3,5-DHBA and Zn^2+^

The scaffold samples were immersed in phosphate buffer solution (PBS) for 1, 3, 5, 7, 10, 14 and 21 days. The immersion solutions were then collected, and the DHBA concentration was quantified using an ultraviolet spectrophotometer at a wavelength of 302 nm. Following the removal of organic matter from the solutions, the Zn^2+^ content was determined via inductively coupled plasma optical emission spectrometry (ICP-OES, Thermo Fisher, USA). Subsequently, the release kinetics of DHBA and Zn^2+^ from the various scaffold samples were fitted to four common kinetic models (zero-order, first-order, Higuchi and Ritger–Peppas) using OriginGraph software (version 8.6).

### Behaviors of Schwann cells cultured in material extracts

#### Preparation of material extracts

Electrospun scaffolds (1, 3 or 5 mg) from each group were soaked in 1 mL of basal culture medium for 3 days at 37°C with 5% CO_2_. According to the initial content of soaking samples, the relevant supernatants (material extract I) were designated as PG-1/3/5, MOF2-1/3/5, MOF4-1/3/5, MOF6-1/3/5. In addition, the presoaked samples were transferred to 1 mL of fresh basal culture medium and incubated for an additional 3 days to obtain material extract II, named as Pre-PG-1/3/5, Pre-MOF2-1/3/5, Pre-MOF4-1/3/5, Pre-MOF6-1/3/5, respectively.

#### Cell viability

Schwann cells (SCs) were purchased from the American Type Culture Collection (ATCC) Biological Standard Resource Center and seeded at a density of 2 × 10^4^ cells/cm^2^ in different material extract I. After 2 and 5 days of incubation, cell viability was evaluated using the Cell Counting Kit-8 (CCK-8) assay. Specifically, a mixture of basal medium (Dulbecco’s Modified Eagle Medium, DMEM) and CCK-8 reagent at a volume ratio of 9:1 was added to each group, followed by incubation for 1 h. Optical density (OD) values were measured at 450 nm using a microplate reader (Bio-Rad 680, USA).

#### Cell morphology

SCs incubated in different material extract I for 2 days were fixed with 4% paraformaldehyde for 30 min. The samples were then incubated with phalloidin dye at 4°C and further stained with 4′,6-diamidino-2-phenylindole (DAPI). Cell morphology was visualized using laser confocal microscopy (Nikon, Japan).

#### Transwell migration assay

Different material extract I (600 μL) was added to the lower chamber of 24-well Transwell inserts (pore size: 8 μm). SCs were resuspended in DMEM, and 300 μL of the cell suspension was seeded into the upper chamber. After incubation for 24 h, cells were fixed with 4% paraformaldehyde solution and stained with 0.05% crystal violet solution for 10 min. Non-migrated cells remaining on the upper surface of the Transwell membrane were carefully removed using a cotton swab. Finally, images of randomly selected fields of view were captured using an inverted phase-contrast microscope.

#### Expression of myelination-related genes

SCs were cultured in the material extract I for 5 days. Total RNA was isolated from the cells using an RNA simple Total RNA Kit (Tiangen Biotech Co., Ltd.) in accordance with the manufacturer’s protocol. First-strand complementary DNA (cDNA) was synthesized using the PrimeScript RT Reagent Kit with gDNA Eraser (Takara Bio Inc.), and quantitative real-time polymerase chain reaction (qRT-PCR) was performed with the SYBR Premix EX Taq Kit (Takara Bio Inc.). The relative expression levels of target genes, including nerve growth factor (*Ngf*), peripheral myelin protein 22 (*Pmp22*) and neural cell adhesion molecule (*Ncam*), were quantified using the 2^-ΔΔ^^*Ct*^ method. Glyceraldehyde-3-phosphate dehydrogenase (*Gapdh*) was selected as the housekeeping gene for data normalization. The sequences of the specific primers used in this study are provided in Table 1.

**Table 1:** Primer sequences related to myelination.

Target genes	Primer sequences
*Ngf*	F: 5′-TGATCGGCGTACAGGCAGA-3′
	R: 5′-GAGGGCTGTGTCAAGGGAAT-3′
*Pmp22*	F: 5′-TGTACCACATCCGCCTTGG-3′
	R: 5′-GAGCTGGCAGAAGAACAGGAAC-3′
*Ncam*	F: 5′-TTCAGTGACGACAGTTCGGAGC-3′
	R: 5′-TGCGAAGACCTTGAGGTGGAT-3′
*Gapdh*	F: 5′-AGGTCGGTGTGAACGGATTTG-3′
	R: 5′-TGTAGACCATGTAGTTGAGGTCA-3′

#### Western blot analysis of PI3K-NGF signaling pathway

Total proteins were extracted from SCs using RIPA lysis buffer, and protein concentrations were quantified with a BCA protein assay kit. Equal amounts of protein samples were separated by 10% SDS-PAGE and transferred onto PVDF membranes. The membranes were blocked with 5% non-fat milk, then incubated overnight with primary antibodies against p-PI3K, PI3K, NGF and GAPDH, respectively. After being washed with TBST buffer, the membranes were incubated with HRP-conjugated secondary antibodies. Protein bands were visualized by an ECL chemiluminescence detection system and recorded for imaging analysis.

### PC12 cell morphology in SC-conditioned medium

For the co-culture assays, SC-conditioned medium was collected as the supernatant after 5 days of direct culture of SCs on the surfaces of different samples. PC12 cells were subsequently cultured in the SC-conditioned medium for 3 days. Afterward, the cells were fixed with 4% paraformaldehyde solution for 30 min and incubated with phalloidin dye at 4°C. Finally, the nuclei were stained with DAPI dye and the samples were observed using a laser scanning confocal microscopy (Nikon, Japan). Neurite length was quantified using ImageJ software for statistical analysis.

### Behaviors of MC3T3-E1 cells cultured in material extracts

#### Cell viability and morphology

MC3T3-E1 cells (purchased from ATCC Biology BioStandard Resource Center) were cultured in material extract I. Cell viability was assessed at 4 and 7 days, and cell morphology was observed at 2 days, following the procedures described in Sections ‘Cell viability’ and ‘Cell morphology’.

#### ALP activity

MC3T3-E1 cells were seeded in well plates at a density of 1 × 10^4^ cells/cm^2^ and cultured with either extract I or material extract II from each group for 4 and 7 days. Afterward, the cells were lysed with a 1% Triton X-100 solution. Equal volumes of the cell lysates were mixed with ALP assay reagents, and the absorbance was measured at a wavelength of 520 nm using a microplate reader. Simultaneously, the total protein concentration of each group was measured using a bicinchoninic acid (BCA) protein assay kit, with absorbance readings recorded at 562 nm. The ALP activity was then calculated by normalizing the ALP-related absorbance values to the corresponding total protein concentrations.

#### Mineralization

MC3T3-E1 cells were incubated with material extract I or material extract II for 14 days. Subsequently, the cells were fixed with 4% paraformaldehyde solution (consistent with prior fixation protocols) and stained with Alizarin Red S (ARS) staining solution for 15–30 min at room temperature. Mineralized nodules formed on the surface were observed under a microscope to capture representative images. For quantitative analysis, cetylpyridinium chloride was added to dissolve the ARS-stained mineralized nodules via incubation for 30 min at 37°C with gentle shaking. The OD value of the resulting solution was measured at a wavelength of 490 nm using a microplate reader.

#### Expression of osteogenesis-related genes

The expression of osteogenesis-related genes, including *Alp*, runt-related transcription factor 2 (*Runx2*) and osteoprotegerin (*Opg*), was evaluated using RT-PCR after 7 days of culture. The primer sequences used for RT-qPCR amplification of target genes and the reference gene (*Gapdh*) are listed in Table 2. The procedure followed the method described in Section ‘Expression of myelination-related genes’.

**Table 2:** Primer sequences related to osteogenesis.

Target genes	Primer sequences
*Alp*	F: 5′-GAACAGAACTGATGTGGAATACGAA-3′
	R: 5′-CAGTGCGGTTCCAGACATAGTG-3′
*Opg*	F: 5′-GCCCAGACGAGATTGAGAG-3′
	R: 5′-CAGACTGTGGGTGACGGTT-3′
*Runx2*	F: 5′-AGAGTCAGATTACAGATCCCAGG-3′
	R: 5′-TGGCTCTTCTTACTGAGAGAGG-3′
*Gapdh*	F: 5′-CTCGTCCCGTAGACAAAATGGT-3′
	R: 5′-GAGGTCAATGAAGGGGTCGTT-3′

### *In vivo* neural injury repair

#### Animals and surgical procedures

Fifty male rats were randomly divided into five groups. All animal experimental procedures were strictly performed in compliance with the ARRIVE guidelines and the National Institutes of Health guide for the care and use of Laboratory animals, and have been approved by the Experimental Animal Ethics Committee of Ruian People’s Hospital (Ethical Review Approval No. SYSQ-2023-21). As shown in Figure 5A, the sciatic nerve was isolated, and a nerve crush injury was induced by alternately clamping the nerve three times at 0.5 cm below the femoral tubercle using a large vascular clamp (clamping duration: 5 s, release interval: 10 s). The resulting 2 mm-wide nerve crush injury was wrapped with different electrospun materials. For the sham operation group, the same surgical exposure and suturing procedures were performed without inducing nerve injury.

#### Sciatic functional index (SFI)

At the 4th and 5th weeks post-surgery, rats were trained to walk along a narrow walkway (60 cm in length, 10 cm in width and 10 cm in height). White paper was laid at the bottom of the walkway, and the hind paws of the rats were coated with black ink. Five measurable footprints were recorded for each rat to calculate the sciatic functional index (SFI) based on the following parameters: print length (PL, distance from the heel to the third toe), toe spread (TS, distance from the first toe to the fifth toe) and intermediate toe spread (IT, distance from the second toe to the fourth toe). Additionally, at the fifth week, the paw extension morphology of the rats was observed and documented through photographs.

#### Electrophysiological assessment

Electrophysiological assessment was conducted at 5 weeks post-surgery to evaluate the recovery of sciatic nerve function using a multi-channel physiological signal acquisition system (MD3000-C, Anhui Zheng Hua Biologic Apparatus Facilities Co., Ltd.). The gastrocnemius muscle and sciatic nerve were exposed and the stimulating electrode was positioned proximal to the injury site to deliver electrical signals. The recording electrode was inserted into the gastrocnemius muscle, the reference electrode was placed on the tendon, and a ground wire was positioned between the stimulating and the recording electrodes. Supramaximal stimulation was applied to elicit compound muscle action potentials (CMAPs).

#### Histological assessment of muscle and nerve fibers

Regenerated nerve and gastrocnemius muscle tissues were isolated and fixed in 4% paraformaldehyde solution for 24 h. After dehydration and clearing, samples were embedded in paraffin. A 30 μm cross-section of the gastrocnemius muscle was stained using Masson’s Trichrome Stain (MTS) Kit, while a 4 μm longitudinal section of the sciatic nerve was stained with hematoxylin–eosin (HE) solution. Additionally, the gastrocnemius muscle tissues were imaged and weighed, and the nerve fibers were observed by transmission electron microscopy.

#### Immunofluorescence staining

To evaluate axon regeneration and remyelination, the expression levels of relevant proteins in longitudinal nerve sections were detected by immunofluorescence staining at 5 weeks post-injury. Sections were incubated with primary antibodies against neurofilament (NF) heavy polypeptide antibody (1:100, Abcam, USA) and myelin basic protein (MBP) (1:10, Abcam, USA), along with DAPI dye. Images were captured using an automatic digital slide scanner (Pannoramic MIDI II, 3DHISTECH Ltd.).

### *In vivo* bone defect repair

An animal model of skull defects was established using 25 female rats. Two defects (each with a diameter of 0.5 cm) were created on both sides of the skull midline, and the defects were subsequently filled with different samples. After 28 days, the skulls were harvested and subjected to micro-computed tomography (micro-CT, Skyscan1176) and HE/Masson staining to visualize new bone formation. Subsequently, bone morphometric parameters, including the relative ratio of bone volume to total volume (BV/TV), the ratio of bone surface area to total volume (BS/TV), trabecular spacing (Tb.Sp) and trabecular number (Tb.N), were analyzed using software packages including CTVox, CTAn and CTVol (Bruker, Germany).

### Statistical analysis

Statistical analysis was conducted using SPSS 25.0 software (SPSS Inc., Chicago, IL). One-way analysis of variance (ANOVA) and Student’s *t*-test were employed to evaluate differences between the groups. Results are presented as mean ± standard deviation (mean ± SD). A confidence level of 95% was used, and differences were considered statistically significant when *P < 0.05.

## Result

### Surface characterization

As shown in the SEM images (Figure 1B), we successfully prepared classical disordered electrospun fibers using Gel and PCL, and deposited a large number of DHBA/Zn-hybrid materials onto the surface via *in situ* synthesis. It was evident that with the increase of Zn^2+^ concentration on the electrospun film, the yield of MOF particles increased significantly. Notably, in the MOF2 group, the DHBA/Zn-hybrid materials nearly covered the entire surface of the electrospun scaffold. Next, the XRD curves (Figure 1C) illustrated the phase composition of the PG, MOF2, MOF4 and MOF6 groups. Two characteristic diffraction peaks at 21.4° and 23.6° were present in all groups, which was consistent with the characteristic peaks of pure PCL/Gel as reported in a previous study [22]. Additional characteristic peaks at 2*θ* values of 8.1°, 19.4°, 22.6°, 25.3°, 26.1°, 28.3° and 36.8° were observed in all the groups except the PG group, indicating the successful incorporation of MOF particles into the electrospun scaffolds. The hydrophilicity of the different groups was evaluated by WCA measurements, with results shown in Figure 1D. The photos reveal a gradient decrease in WCA values, indicating an improvement in the wettability of the materials with the increasing deposition of DHBA/Zn-hybrid materials. The detailed WCA values are as follows: 65.5 ± 5.7° (PG), 42.0 ± 5.4° (MOF2), 27.3 ± 0.9° (MOF4), 24.5 ± 1.8° (MOF6).

**Figure 2:**
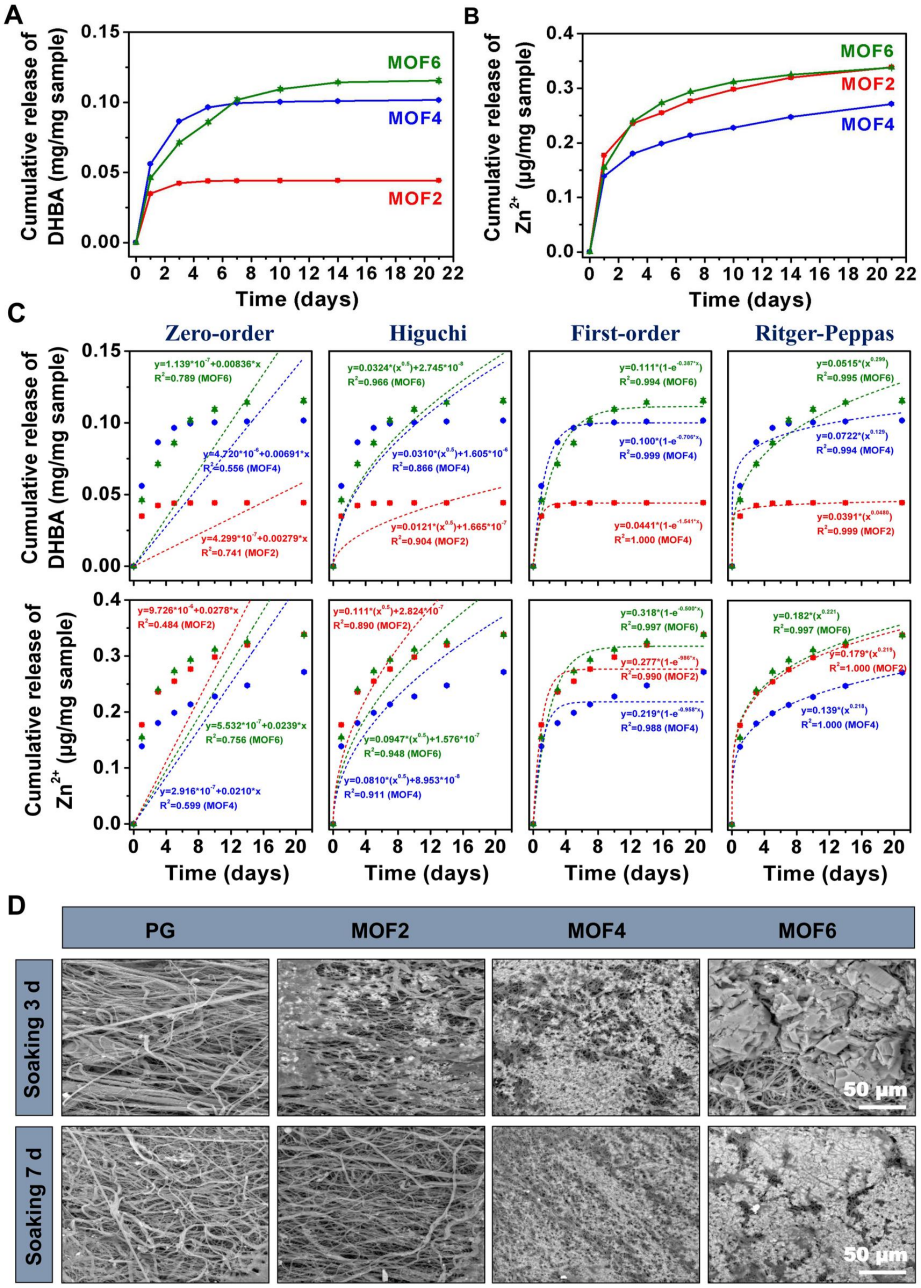
Sustained-release profiles of (**A**) DHBA and (**B**) Zn^2+^ from different electrospun scaffolds over a 21-day period; (**C**) release kinetics of DHBA and Zn^2+^ from various samples; (**D**) SEM images depicting surface morphology changes of electrospun scaffolds after immersion in PBS solution for 3 and 7 days.

To investigate the degradation behavior of the DHBA/Zn-hybrid materials, the release profiles of DHBA and Zn^2+^ were measured. As shown in Figure 2A, the MOF2 group exhibited the lowest DHBA release, with nearly complete release achieved by day 3 (0.042 mg/mg sample). In contrast, the MOF4 group showed a significantly higher release rate, reaching 0.096 mg/mg sample by day 5, followed by a gradual slowdown. The MOF6 group demonstrated a slightly lower DHBA release than the MOF4 group within the first 7 days, but surpassed the MOF4 group thereafter. The release rate began to decelerate after day 14, reaching 0.114 mg/mg sample. The initially slower release in the MOF6 group may be attributed to its higher MOF content and more stable structure. However, its larger initial DHBA loading capacity contributed to an extended-release period and the highest total release amount within 21 days. SEM images of the different electrospun scaffolds soaked in PBS for 3 and 6 days (Figure 2D) further confirmed a substantial reduction in the content of DHBA/Zn-hybrid materials across all the groups. The MOF2 group was largely degraded by day 3 and had completely disappeared by day 7. In contrast, a significant amount of material remained on the surfaces of the MOF4 and MOF6 scaffolds (especially the latter) by day 7, indicating their potential for long-term sustained release. Additionally, as shown in Figure 2B, all the three groups exhibited sustained and slow release of Zn^2+^ over 21 days. Among them, the MOF4 group showed the slowest release rate, while the MOF2 and MOF6 groups displayed similar release behaviors. By day 21, the final Zn^2+^ release concentrations of the MOF2, MOF4 and MOF6 groups were 0.34, 0.27 and 0.34 μg/mg sample, respectively. The slower Zn^2+^ release in the MOF4 group may be attributed to the formation or adsorption of more DHBA/Zn-MOF or free DHBA on the fiber surface. The similar release pattern observed in the MOF6 and MOF2 groups could result from the combined effects of higher initial Zn^2+^ loading and surface coverage by DHBA/Zn-MOF or free DHBA. Further analysis of the release kinetics revealed that the DHBA release profiles in the MOF2 and MOF4 best fit the first-order model (*R*^2^ = 0.999 and 1.000, respectively), whereas the MOF6 group followed the Ritger–Peppas model more closely (*R*^2^ = 0.995, *n* = 0.299). In contrast, the Zn^2+^ release in all the three groups was well described by the Ritger–Peppas model (*R*^2^ = 1.000, 1.000 and 0.997; *n* = 0.219, 0.218 and 0.221, respectively). Since all *n* values were below 0.45, the Zn^2+^ release was determined to follow a Fickian diffusion mechanism.

### SC behaviors

**Figure 3:**
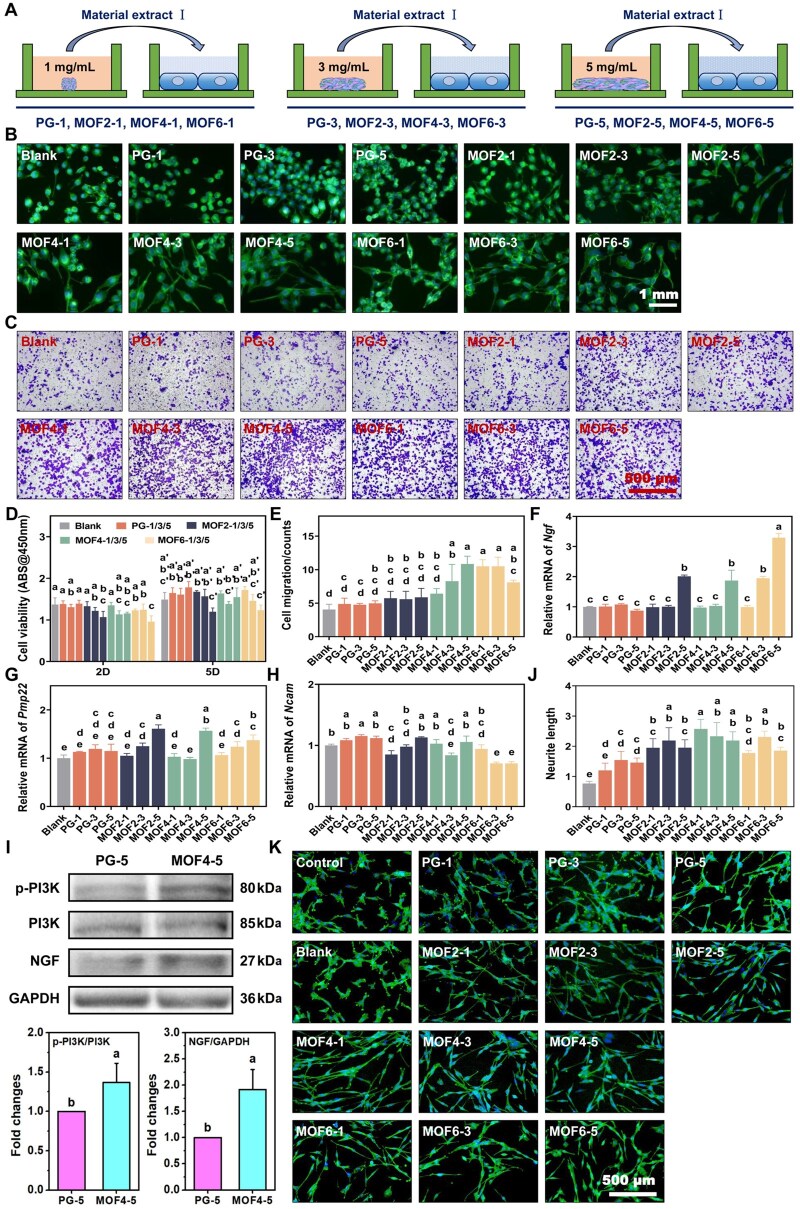
(**A**) Schematic illustration of the preparation process of material extract I and grouping for cell-based assays; (**B**) representative images depicting the morphology of Schwann cells (SCs) cultured in different material extracts; (**C**) representative images of SC migration observed across the experimental groups; (**D**) quantitative analysis of SC viability in each group; (**E**) statistical data on the number of migrating SCs; relative expression levels of neural-specific genes, including (**F**) *Ngf*, (**G**) *Pmp22* and (**H**) *Ncam*, in SCs; (**I**) WB images and quantitative analysis of PI3K-NGF signaling pathway-related proteins; (**J**) representative images illustrating axonal extension and (**K**) cell morphology of PC12 cells exposed to the SC-conditioned medium. Groups labeled with identical letters (**a**–**e**, **a**′–**c**′) indicate no significant differences, whereas distinct letters signify statistically significant differences (*P *< 0.05).

After incubation with in different material extract I (Figure 3A), SCs in the Blank group and the PG-1/3/5 groups appeared a nearly round shape (Figure 3B). Starting from the MOF2-1 group, cells gradually adopted a spindle shape with distinct protrusions. The protrusions in the MOF4-1/3/5 groups were significantly longer than those in the other groups, followed by the MOF6-1/3/5 groups. The cell viability results (Figure 3D) revealed that on day 2, groups with high Zn^2+^ concentrations, such as MOF2-5, MOF4-5 and MOF6-5, exhibited relatively lower cell viability compared to the blank group (cells cultured in normal medium). By day 5, the PG-1/3/5, MOF2-1/3, MOF4-1 and MOF6-1 groups demonstrated a slight increase in cell viability relative to the Blank group. In contrast, MOF2-5, MOF4-3 and MOF6-5 showed obvious cytotoxicity, though the inhibition of cell activity remained within an acceptable range. Overall, the materials exhibited good biocompatibility, with only minor inhibitory effects observed in the high-concentration groups.

For cell migration, Figure 3C shows that the blank group and the PG-1/3/5 groups exhibited minimal vertical cell migration. After DHBA/Zn-modification, SC migration activity was significantly enhanced, with the MOF4 and MOF6 groups outperforming the MOF2 group. Quantitative analysis (Figure 3E) revealed that at initial immersion concentrations of 1 and 3 mg/mL, cell migration ability increased progressively with higher MOF content in the samples (MOF6-1 > MOF4-1 > MOF2-1; MOF6-3 > MOF4-3 > MOF2-3). However, at a concentration of 5 mg/mL, the MOF4 group exhibited the most pronounced enhancement of cell migration, while the MOF6 group displayed a slight decline compared to MOF4 but still outperformed the MOF2 group (MOF4-5 > MOF6-5 > MOF2-5). In summary, the materials demonstrated the capacity to enhance cell migration, with MOF4-5 showing the most remarkable performance.

The expression of myelination-related genes in SCs, including *Ngf*, *Pmp22* and *Ncam*, was assessed by RT-qPCR. As shown in Figure 3F, *Ngf* expression was significantly (*P *< 0.05) upregulated in the MOF2-5, MOF4-5 and MOF6-3/5 groups, with MOF6-5 showing the highest increase compared to the blank group. For *Pmp22* (Figure 3G), all the experimental groups showed varying degrees of upregulation compared to the blank group, with MOF2-5 and MOF4-5 displaying the most pronounced effects. In contrast, *Ncam* expression (Figure 3H), which is associated with pre-myelination, was significantly (*P *< 0.05) downregulated in the MOF4-3 and MOF6-3/5 groups. Overall, the experimental groups demonstrated enhanced expression of myelination-related genes and neurotrophic factors, with the 5 mg/mL concentration groups achieving the most notable results. This alteration in gene expression pattern suggests that the material at this concentration may be more conducive to the transformation of SCs toward a myelinating phenotype.

To further elucidate the molecular mechanism underlying the neuroregenerative effect of DHBA/Zn-MOF scaffolds, we conducted WB analysis for the preliminary validation of key proteins in the PI3K-NGF signaling pathway. Based on the *in vitro* cell migration and gene expression results, the MOF4-5 group (exhibiting the optimal neuro-promotive effects) and the PG-5 group (control, without DHBA/Zn-MOF modification) were selected for comparative analysis. The results (Figure 3I) showed that compared with the PG-5 group, the phosphorylation levels of p-PI3K were significantly upregulated by approximately 1.4-fold in the MOF4-5 group, and the protein expression level of NGF was further elevated by approximately 1.9-fold. These results directly confirm that DHBA/Zn-MOF composite scaffold activates the PI3K-NGF signaling pathway to promote SC myelination and neural regeneration, serving as the core mechanism identified by our preliminary mechanistic validation for the material’s neuroregenerative function.

### PC12 cell behaviors

The results in Figure 3J and 3K illustrate the effects of different materials on the morphology and neurite outgrowth of PC12 cells cultured in SC-conditioned medium. Fluorescence observations (Figure 3K) revealed no significant neurite formation in the control (normal medium) and blank groups (supernatant of SCs cultured in normal medium). However, starting from the PG-1 group, cell morphology in the experimental groups became increasingly elongated, with progressive neurite extension. Quantitative analysis of neurite length (Figure 3J) showed that at initial immersion concentrations of 1, 3 and 5 mg/mL, the MOF4 group exhibited the longest neurites. Although the MOF6 group displayed slightly shorter neurites than the MOF4 group, it still outperformed the MOF2 and PG groups (MOF4 > MOF6 > MOF2 > PG). Collectively, the SC-conditioned medium from each experimental group promoted neurite outgrowth of PC12 cells to varying degrees, with the MOF4 exhibiting the most prominent inductive effect. Combined with the above experiments, the target material effectively induced SCs to secrete cytokines including Ngf, thereby indirectly facilitating the differentiation of PC12 cells into cells with neuronal morphology, a key structural prerequisite for subsequent functional neuronal regeneration. Notably, neurite outgrowth, as a core histological marker of neuronal differentiation, provides morphological evidence for the potential of the scaffold to support neural function recovery rather than direct proof of functional regeneration itself.

### MC3T3-E1 cell behaviors

**Figure 4:**
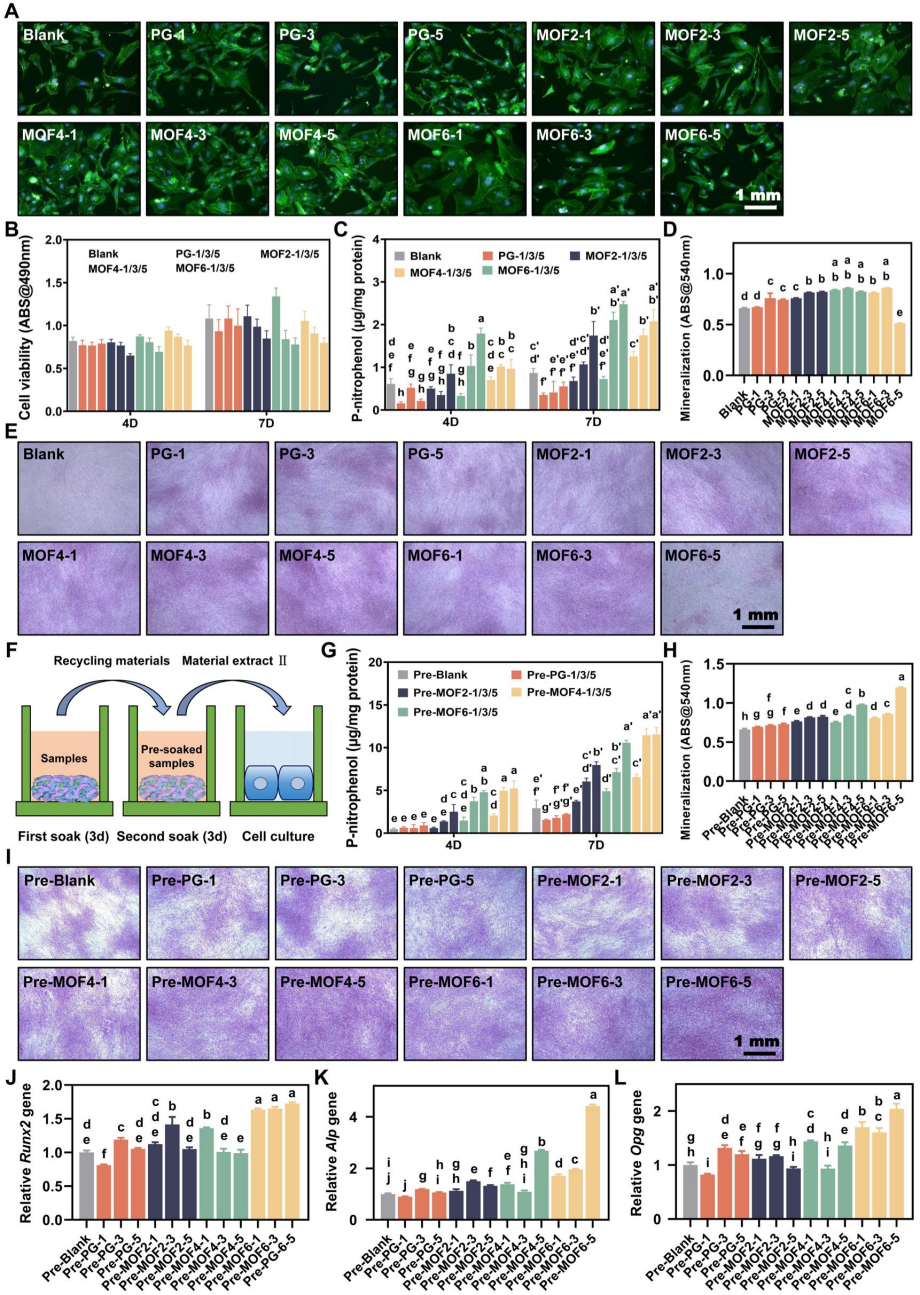
(**A**) Representative images of MC3T3-E1 cell morphology cultured in different extracts; (**B**) cell viability of MC3T3-E1 cells across all experimental groups; (**C**) alkaline phosphatase (ALP) activity and (**D**) mineralization levels of MC3T3-E1 cells in different groups; (**E**) representative images of mineralized nodule staining in MC3T3-E1 cells; (**F**) schematic diagram illustrating the preparation process of material extract II, wherein samples were presoaked for 3 days; (**G**) ALP activity, (**H**) mineralization levels and (**I**) mineralized nodules for MC3T3-E1 cells in the presoaked experimental groups; relative expression levels of osteogenic markers, including (**J**) *Runx2*, (**K**) *Alp* and (**L**) *Opg*, in MC3T3-E1 cells from the presoaked experimental groups. Groups labeled with identical letters (**a**–**i**, **a**′–**g**′) indicate no significant differences, while distinct letters signify statistically significant differences (*P *< 0.05).

To evaluate the osteogenic properties of various materials, extracts were prepared from samples at different concentrations on day 3 (material extract I) and day 6 (material extract II) under identical conditions. The morphology of MC3T3-E1 cells cultured in material extract I is shown in Figure 4A. Cells in the MOF2-1/3/5, MOF4-1/3/5 and PG-6-1 groups displayed slightly increased spreading compared to the blank group. As for cell viability (Figure 4B), on both day 4 and day 7, the 5 mg/mL groups exhibited obvious cytotoxicity, while the 1 mg/mL groups demonstrated slightly higher cell viability than the blank group. Notably, the MOF6-1 and MOF4-1 groups on day 4 and day 7 showed markedly enhanced cell viability, respectively. ALP activity (Figure 4C) analysis revealed that on day 7, the MOF2-5, MOF4-3/5 and MOF6-3/5 groups exhibited significantly (*P *< 0.05) higher ALP activity compared to the blank group. On day 4, only the MOF4-3/5 and MOF6-3/5 groups showed significantly elevated ALP activity. Alizarin red staining (Figure 4E) indicated a significant increase in mineralized nodules in all the experimental groups except MOF-5 compared to the blank group. Quantification of mineralization (Figure 4D) further confirmed these observations, showing that the PG-3/5, MOF2-1/3/5, MOF4-1/3/5 and MOF6-1/3 groups had significantly (*P *< 0.05) enhanced mineralization compared to the blank group, while the MOF6-5 group showed a significant decrease (*P *< 0.05).

For material extract II (Figure 4F), ALP activity data (Figure 4G) demonstrated that the Pre-MOF4-5 and Pre-MOF6-3/5 groups exhibited the highest ALP activity at both day 4 and day 7 among all the groups. Additionally, the Pre-MOF2-3/5, Pre-MOF4-1/3 and Pre-MOF6-1 groups showed significantly (*P *< 0.05) higher ALP activity compared to the blank group on day 7, whereas only the Pre-MOF2-5, Pre-MOF4-3 and Pre-MOF6-1 groups exhibited elevated activity on day 4. Alizarin red staining and its quantification (Figure 4H and I) revealed that Pre-MOF6-5 had the highest mineralized nodule formation among all the groups. Other groups, including Pre-PG-1/3/5, Pre-MOF2-1/3/5, Pre-MOF4-1/3/5 and Pre-MOF6-1/3, also showed significantly increased mineralization compared to the blank group. Gene expression analysis (Figure 4J–L) further highlighted that MOF6-5 had the highest expression levels of *Runx2*, *Alp* and *Opg* genes, key molecular markers of osteogenic differentiation. These elevated gene expressions provide molecular evidence supporting the scaffold’s potential to promote osteogenic regeneration, which is consistent with the subsequent *in vivo* functional bone repair observed in micro-CT and histological assessments. Overall, these findings suggest that the osteogenic-promoting effects of MOF4 were most pronounced when the material extract was directly applied. However, after presoaking for 3 days, the MOF6 material exhibited the optimal effect, potentially due to the degradation of a substantial portion of MOF from the material surface.

### Repair of sciatic nerve injury

**Figure 5:**
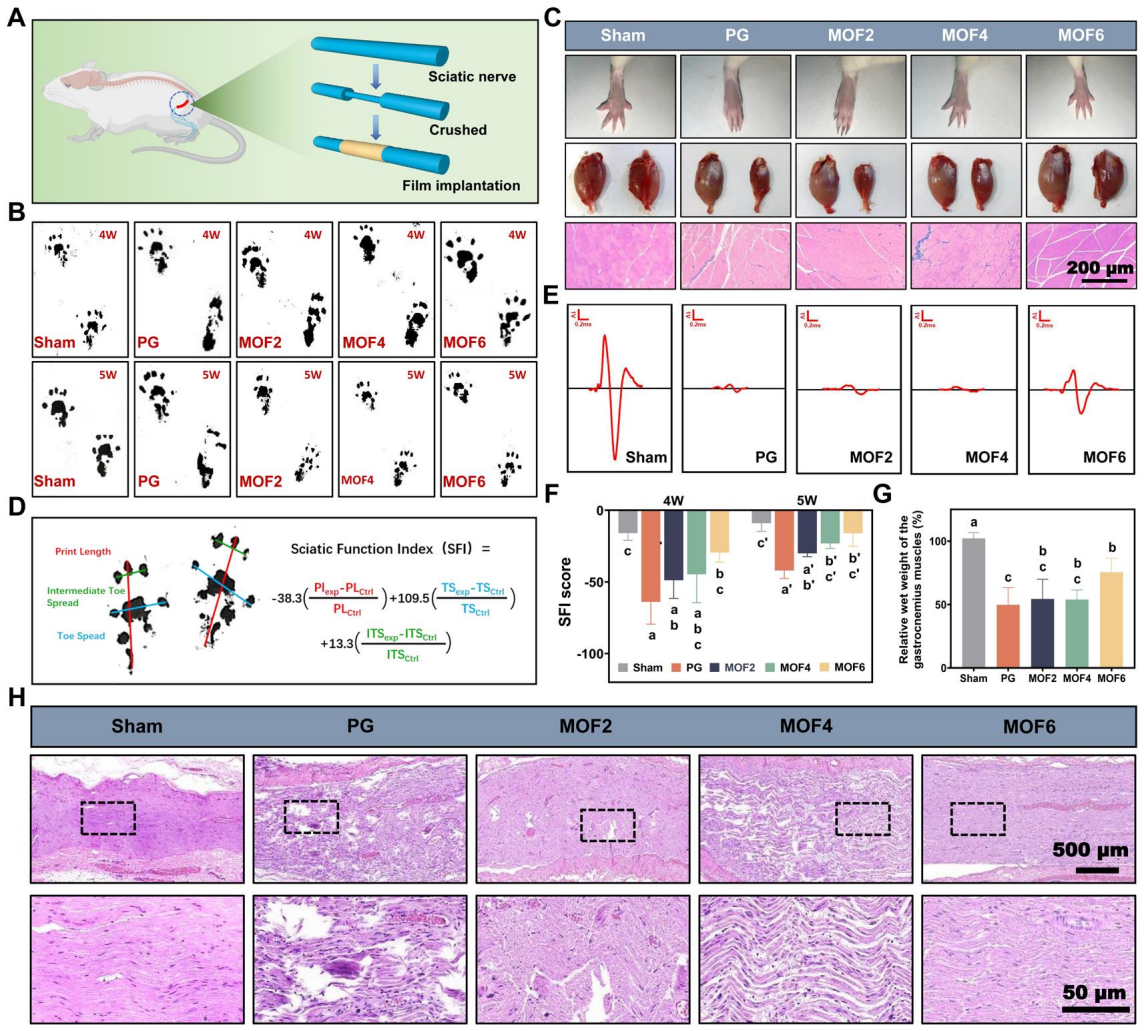
(**A**) Schematic representation of electrospun film implantation at the sciatic nerve defect site; (**B**) footprint patterns of rat hindlimbs recorded at 4 and 5 weeks post-surgery; (**C**) representative images showcasing the hindlimb toes, gastrocnemius muscle and histological staining of the gastrocnemius muscle across experimental groups; (**D**) schematic depiction of the calculation method for the sciatic functional index (SFI); (**E**) compound muscle action potential (CMAP) recordings of the gastrocnemius muscle in each group; (**F**) quantitative analysis of SFI values with statistical evaluation; (**G**) quantitative analysis of gastrocnemius muscle weight at 5 weeks post-surgery; (**H**) hematoxylin and eosin (HE) staining images of sciatic nerve tissues in each group. Groups labeled with identical letters (**a**–**c**, **a**′–**c**′) indicate no significant differences, while distinct letters signify statistically significant differences (*P *< 0.05).

A rat sciatic nerve crush injury model was established to evaluate the effect of various materials on nerve repair, focusing on neurological function indices, gastrocnemius muscle recovery, electrophysiological properties and histological analysis. Footprint experiments were performed at 4 and 5 weeks post-surgery, and the results are shown in Figure 5B. At week 4, the PG and MOF2 groups exhibited longer footprints and a shorter inter-toe distance, while the MOF4 and MOF6 groups displayed slightly shorter footprints and toe spreading. These observations were further corroborated by optical images of the rat toes (Figure 5C). By week 5, the footprints of the MOF2, MOF4 and MOF6 groups showed significant improvement, with the MOF6 group closely resembling the sham group. Correspondingly, SFI values calculated from these results indicated that after 4 weeks of treatment with electrospun dressings. The SFI was calculated as shown in Figure 5D, where EPL, ETS and EIT represent the experimental values of the aforementioned parameters, and NPL, NTS and NIT represent the normal values. Motor function recovery in the experimental groups was superior to that in the control group (score = −63.93 ± 12.71; Figure 5F). A concentration-related improvement was observed with increasing MOF concentrations, particularly in the MOF6 group (score = −29.48 ± 5.75). By week 5, the SFI score of the MOF6 group (−16.15 ± 7.74) approached that of the sham group (−9.00 ± 4.70), consistent with the footprint observations.

Additionally, photographs were taken of the gastrocnemius muscles from both the affected and unaffected sides of rats in each group. As shown in Figure 5C, the size of the gastrocnemius muscle on the affected side in the MOF6 group was nearly identical to that on the normal side, while the gastrocnemius muscles in the PG, MOF2 and MOF4 groups remained atrophied. Histopathological analysis (Figure 5C) also revealed that the muscle fibers in the MOF6 group exhibited regular morphology and texture, in contrast to the disorganized fiber structure observed in the PG group. Further analysis of the wet weight of the affected gastrocnemius muscle (Figure 5G) revealed a significant increase in the wet weight ratio in the MOF6 group when compared with the PG group. The CMAP of the gastrocnemius muscle was also measured in this study. CMAP amplitude is positively correlated with the density of regenerated nerve fibers passing through the injury site and innervating the target muscle. As shown in Figure 5F, the CMAP amplitudes in the PG, MOF2 and MOF4 groups were significantly reduced, whereas the CMAP waveform in the MOF6 group gradually aligned with that of the sham group, indicating better nerve fiber regeneration and target muscle innervation in the MOF6 group.

**Figure 6:**
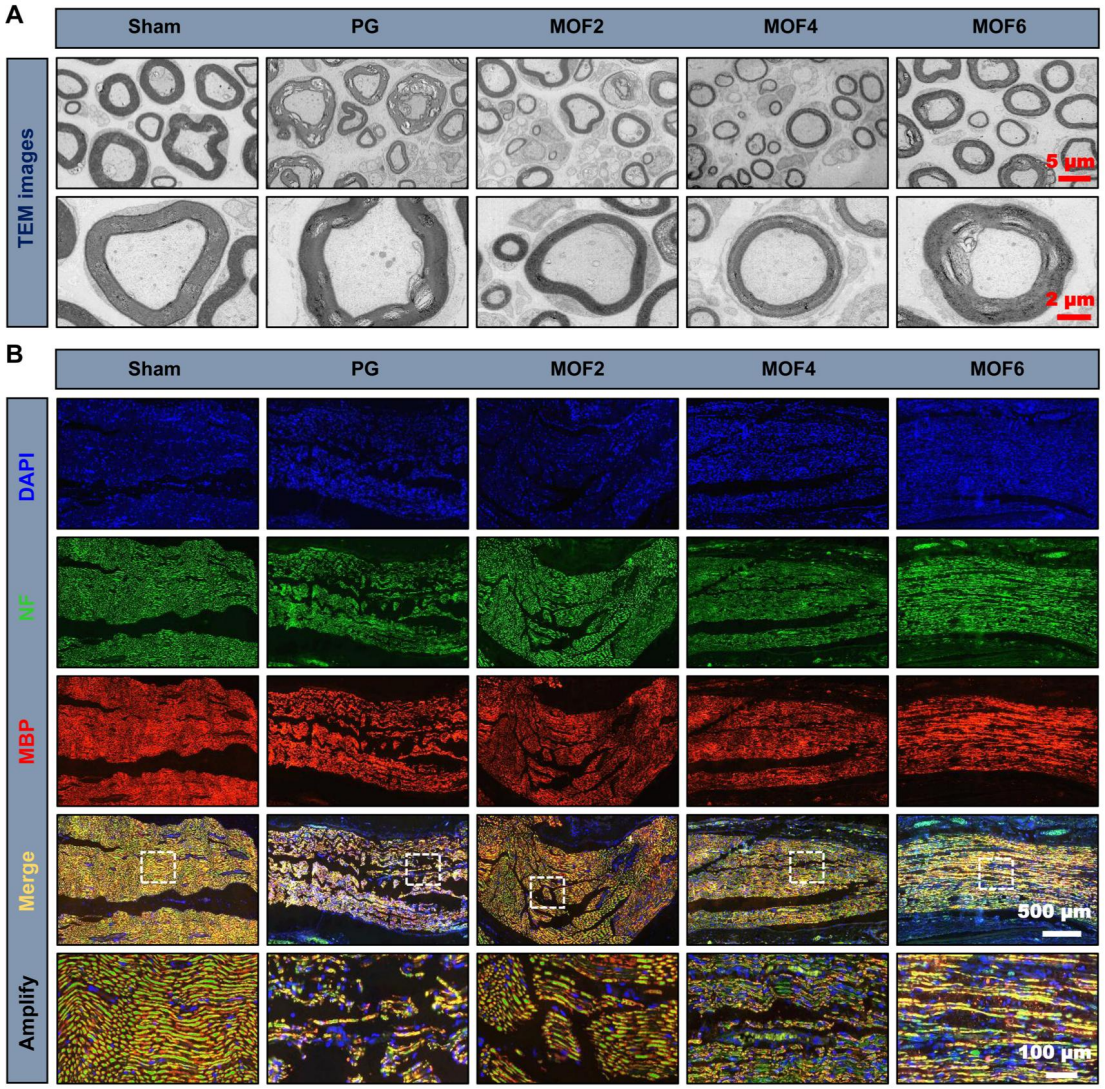
(**A**) Transmission electron microscopy (TEM) images of sciatic nerve tissue; (**B**) fluorescent staining images of sciatic nerve tissues depicting 4′,6-diamidino-2-phenylindole (DAPI), neurofilament (NF) and myelin basic protein (MBP) expression in the different groups.

The above results have confirmed that the target material can effectively improve the sensory and motor functions of the injured nerve, thereby achieving functional regeneration. There is an extremely close relationship between the structure and function of nerves, summarized by the principle that the degree of functional recovery depends entirely on the quality of structural repair. This relationship is particularly evident in peripheral nerves such as the sciatic nerve. Therefore, we conducted histological analyses of the injured nerve to investigate its structural changes. The results in Figure 5H indicate that the use of MOFs significantly improved the axonal fiber tissue structure at the injury site. In the MOF6 group, the axonal fibers were well-organized and demonstrated excellent recovery, resembling those in the uninjured sham group. In contrast, the nerve fiber structure in the PG group was sparse and disordered, indicating severe damage. TEM images of the sciatic nerve (Figure 6A) further supported this observation. The sciatic nerve in the PG group exhibited a loose and disorganized state, while the regenerated axons in the MOF6 group were surrounded by distinct and thick myelin sheaths, a critical histological marker closely correlated with the recovery of nerve conduction function (as verified by CMAP measurements in functional assessments). Thicker and well-organized myelin sheaths reduce ion leakage during nerve impulse transmission, which serve as the structural basis for the improved motor function (e.g. SFI, footprint morphology) observed in the MOF6 group. In addition, NF and MBP staining were used to effectively assess the formation of axons and regenerated myelin sheaths, with NF protein representing regenerated neurofilaments and axons, and MBP indicating myelinated fibers. According to the DAPI/NF/MBP fluorescence staining images (Figure 6B), the PG group exhibited a disorganized structure with no continuous long axons formed. However, the MOF6 group demonstrated continuous axons, indicating the successful formation of long axons. Additionally, MBP-stained cells in the MOF6 group were aligned along the long axons, further confirming the presence of regenerated myelinated nerve fibers at the injury site.

### Repair of bone defect

**Figure 7:**
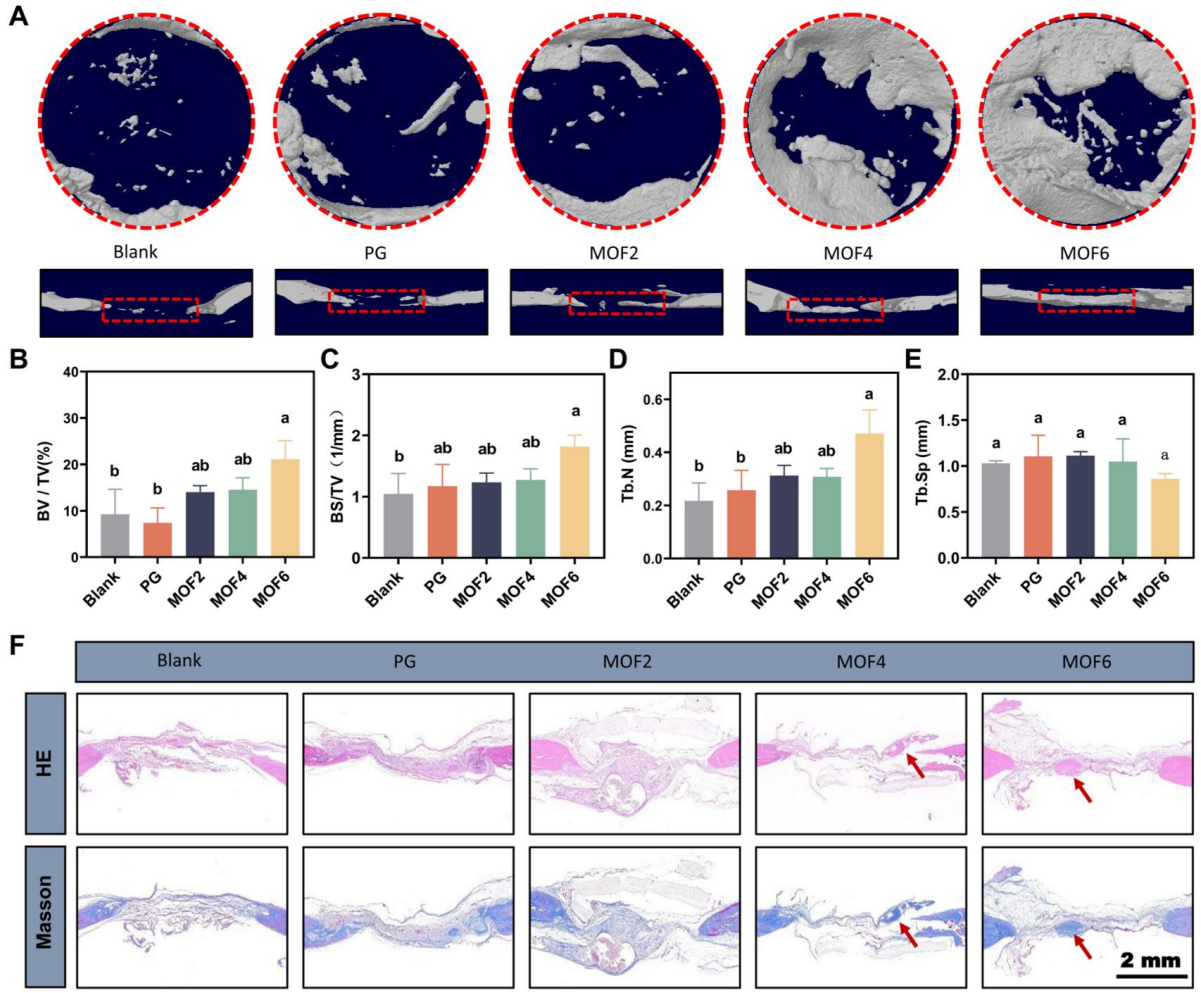
(**A**) Three-dimensional reconstructed images of rat cranial bone in coronal and sagittal views across all the groups; quantitative analysis of bone volume to total volume (BV/TV, **B**), the ratio of bone surface area to total volume (BS/TV, **C**), trabecular number (Tb.N, **D**) and trabecular spacing (Tb.Sp, **E**); (**F**) histological assessment of sciatic nerve tissues via hematoxylin and eosin (HE) staining and Masson staining in each group. Groups labeled with identical letters (**a**, **b**) indicate no significant differences, while distinct letters signify statistically significant differences (*P *< 0.05).

To further assess the osteogenic potential of these materials, skull defect experiments were conducted. Micro-CT imaging and subsequent 3D reconstructions (Figure 7A) enabled visual comparison of skull defect repair among the experimental groups. Both the PG group and the control group exhibited prominent bone defect areas with minimal repair. However, the MOF6 group displayed a significant reduction in the defect area, underscoring its prominent structural bone regeneration potential. Quantitative analysis of bone microstructural parameters, including BV/TV (Figure 7B), BS/TV (Figure 7C), Tb.N (Figure 7D) and Tb.Sp (Figure 7E), revealed trends consistent with these observations. Furthermore, histological evaluations using HE and Masson staining (Figure 7F) illustrated that the MOF4 and MOF6 groups, particularly the latter, exhibited increased formation of new bone (indicated by arrows) within the bone defect region. In summary, these findings, supported by micro-CT, histological staining and bone microstructural parameters, highlight that the MOF6 group exhibits superior structural bone regeneration potential, which provides a critical morphological basis for functional bone repair (e.g. restored load-bearing capacity). Its osteogenic capabilities, verified by both structural and molecular evidence, significantly exceed those of the control group.

## Discussion

In this study, DHBA/Zn-MOF nanoparticles were successfully embedded into Gel/PCL electrospun scaffolds using a wet electrospinning technique [29, 30]. SEM analysis of the control group revealed a typical disordered electrospun structure, consistent with findings by Guner *et al.*, who observed similar architectures in Gel/PCL-based scaffolds fabricated under comparable conditions [31]. Through an *in situ* coordination reaction between Zn^2+^ and DHBA, morphologically regular particles were observed to grow along the surface of the electrospun scaffold. The extensive interaction between the electrospun fibers and the receiving liquid (ethanol/DHBA solution) facilitated a high MOF loading, resulting in a scaffold surface that appeared entirely coated with MOF particles [32]. This study also revealed that increasing Zn^2+^ concentrations led to a gradual enlargement of MOF particles, likely due to excessive particle production and subsequent fusion. Elemental mapping further demonstrated that in Zn^2+^-enriched regions, the formation of numerous MOF structures resulted in their aggregation into larger, elongated particles along the electrospun fibers. Yang *et al*. previously developed a bipolar metal-flexible electrospun fibrous membrane incorporating MOFs, utilizing them as carriers for the sustained release of metal ions while promoting osteogenesis and tendon formation on the scaffold [33]. Similarly, this study utilized a delivery system integrating electrospun scaffolds and MOFs to achieve sustained release of DHBA and Zn^2+^, as further validated by SEM analysis post-release. Additionally, XRD analysis identified characteristic PCL/Gel peaks, aligning with previous studies on Gel/PCL composites [22]. Compared with the PG group, the MOF2/4/6 groups exhibited new peaks at 2*θ* values of 13.9°, 19.3°, 22.5°, 25.1°, 28.1° and 36.8°, which were similar to the crystalline profile of MOF-74 as described by Dietzel *et al.* [34]. These findings collectively confirm the successful *in situ* synthesis of MOF particles on the PG electrospun scaffold through a coordination reaction between DHBA and Zn^2+^.

To evaluate the biological behavior of SCs, we examined the effects of material extracts on SC proliferation, migration and gene expression. SCs, as specialized myelinating glial cells, are indispensable for peripheral nerve regeneration following traumatic injury and neuropathies [35, 36]. After nerve injury, SCs lose contact with axons at the distal stump and undergo demyelination, transitioning into a repair phenotype. This phenotype plays a crucial role in Wallerian degeneration, facilitating the disintegration of damaged axons and the clearance of myelin debris [37]. Our results demonstrate that DHBA/Zn-MOF materials significantly accelerate the migration rate of SCs, creating a favorable microenvironment for nerve repair. This enhanced migration is hypothesized to expedite the clearance of inhibitory debris and enable faster axonal regeneration. Additionally, repair-phase SCs secrete essential neurotrophic factors, including NGF, neurotrophin-3 (NT3) and brain-derived neurotrophic factor (BDNF), which support neuronal survival and promote axonal outgrowth [38, 39]. To delve deeper into the molecular mechanisms, we used real-time fluorescence PCR to analyze the expression of *Ngf*, *Pmp22* and *Ncam* genes, which collectively reflect the SCs’ transition from a repair to a myelinating state. Immature SCs are characterized by high expression of NCAM and p75 during development [40]. However, during the myelination phase, NCAM expression is downregulated while myelin-associated proteins, such as PMP22, are upregulated [40, 41]. This switch not only supports myelin regeneration but also orchestrates the guidance and elongation of regenerating axons. Remarkably, our findings confirm that the MOF-modified materials promote myelin regeneration, thereby accelerating nerve repair. This process is primarily driven by the synergistic effects of DHBA and Zn^2+^. DHBA, a unique compound with neuroregenerative potential, played a pivotal role in enhancing SC behavior. The neurotrophic effects of DHBA may result from its structural resemblance to protocatechuic acid, a compound known to stimulate SC proliferation and migration via the IGF-IR-PI3K signaling pathway [42]. For the preliminary validation of the core molecular mechanism of our scaffold’s neuroregenerative action, we performed WB analysis of the PI3K-NGF pathway, a classical cascade that mediates NGF-induced neuroregeneration [43]. The results confirmed that DHBA/Zn-MOF scaffolds significantly upregulated p-PI3K/PI3K in SCs, accompanied by increased NGF protein expression (Figure 3I). This directly confirms activation of the PI3K-NGF pathway, which aligns with our RT-qPCR findings of elevated *Ngf* gene expression, forming a ‘gene–protein’ evidence chain for our preliminary mechanistic conclusions. Notably, PI3K has long been recognized as indispensable for neuronal survival, differentiation and SC functional regulation [43]. Zn^2+^ further potentiates this pathway by stabilizing the interaction between PI3K and its upstream activators, inhibiting phosphatase-mediated dephosphorylation of PI3K to prolong NGF secretion and signaling activation [44, 45]. Collectively, the synergistic activation of the PI3K-NGF signaling pathway by DHBA and Zn^2+^ is the key molecular mechanism revealed by our preliminary experimental validation, which underlies the enhanced SC myelination capacity and robust neuroregenerative efficacy of DHBA/Zn-MOF scaffolds.

Wound healing around implanted materials is a complex biological process. Upon implantation, rapid protein adsorption triggers the recruitment of medullary cells, such as monocytes and macrophages, which regulate the inflammatory response by releasing cytokines and chemokines [46, 47]. Within 3–5 days, mesenchymal stem cells and other repair cells are recruited to the site, initiating tissue regeneration [48]. Current research suggests that the initial cytotoxicity of biomaterials has minimal impact on long-term repair outcomes, as it occurs before significant tissue repair cell recruitment and activity [48, 49]. Our study aligns with these findings, showing that while MOF6 electrospun fillers exhibit early cytotoxicity toward osteoblasts *in vitro*, this does not impede their overall ability to promote tissue repair. After a 3-day prerelease period, material extracts demonstrated significant enhancements in osteoblast differentiation, ALP activity and mineralization levels. Importantly, long-term *in vivo* studies revealed consistent osteogenic trends, with the MOF6 group achieving optimal bone regeneration outcomes, supported by micro-CT imaging and histological analysis. For neuroregeneration, *in vivo* experiments confirmed that MOF6 scaffolds significantly enhanced nerve fiber remyelination and axonal regeneration. The MOF6 group outperformed other groups in functional recovery, as evidenced by improved electrophysiological signals, footprint tests, and histological organization. Immunofluorescence staining further validated the high expression of key markers, such as NF and MBP, indicating robust neural repair processes [50, 51]. For osteogenesis, micro-CT imaging and histological analysis confirmed the MOF6 group achieved optimal bone regeneration in the cranial defect model. While this study focuses on the dual-functional repair of peripheral nerve injury and bone defects (a common clinical challenge in oral and maxillofacial surgeries, trauma and other fields), we selected sciatic nerve crush injury and cranial defect models for their standardized protocols, reliable quantitative indexes (SFI, CMAP, BV/TV) and accessibility. These models enable objective, reproducible evaluation of neural functional recovery (motor function, electrophysiology) and bone structural regeneration (micro-CT parameters, histological staining), providing critical preliminary validation of the scaffold’s core efficacy. Nearly all bone regeneration involves coordinated repair of hard tissue and neural networks. Sensory neurons regulate bone homeostasis via neuropeptides, and functional bone regeneration supports neural reinnervation. Thus, the DHBA/Zn-MOF scaffold’s dual-functional mechanism (synergistic neuroregeneration and osteogenesis) is not limited to alveolar socket repair. It holds broad translational potential in neuro-bone composite injury scenarios, such as traumatic craniofacial defects, fractures with peripheral nerve injury and osteoporotic fractures with neurovascular impairment. Its core design integrates neurotrophic and osteogenic components into a sustained-release electrospun system, offering a novel strategy for synergistic regeneration of interconnected tissues. Notably, the mechanisms of peripheral nerve regeneration (e.g. SC activation, myelin repair, axonal outgrowth) and bone regeneration (e.g. osteoblast differentiation, mineralization) are conserved across different anatomical sites. Thus, the DHBA/Zn-MOF scaffold’s dual-functional mechanism is not limited to a specific region but can be translated to neuro-bone composite injury scenarios, including oral and maxillofacial surgeries, traumatic craniofacial defects and fractures with peripheral nerve involvement.

In summary, the MOF6 electrospun scaffolds demonstrated excellent efficacy in promoting nerve repair and regeneration. These findings not only validate the functional design of MOF materials but also contribute to the growing evidence supporting their application in nerve tissue engineering. Despite the promising results, this study has certain limitations. First, we have not constructed an oral and maxillofacial-specific neuro-bone composite injury model (e.g. alveolar socket defect combined with inferior alveolar nerve injury) to verify the material’s performance in clinical scenarios such as impacted tooth extraction. Key constraints include the narrow anatomy of the mandibular canal (hindering standardized injury induction and scaffold implantation) and the lack of objective quantification for sensory function evaluation in small animals. Future studies will address this by optimizing a large-animal model (e.g. cone-beam CT-guided localization of the inferior alveolar nerve and defect creation) to simulate oral and maxillofacial surgeries, directly validating the scaffold’s applicability in this specific clinical setting. Second, the molecular mechanisms underlying the scaffold’s regulation of neural and osteogenic cellular behaviors remain incompletely understood. Further research is required to elucidate the pathways involved, such as those mediating SC repair phenotypes or osteoblast mineralization. Additionally, the long-term stability and degradation of MOF scaffolds *in vivo* require comprehensive evaluation to ensure their safety and efficacy for clinical applications.

## Conclusion

In this study, we developed an innovative series of multifunctional electrospun scaffolds by incorporating DHBA/Zn-MOF particles into a Gel/PCL matrix via *in situ* synthesis within DHBA solutions. The resulting MOF-doped scaffolds (MOF2, MOF4 and MOF6) exhibited significantly enhanced hydrophilicity compared to PG scaffolds. Among these, MOF-modified scaffolds (especially MOF4) demonstrated superior performance in promoting neural growth and osteogenic differentiation *in vitro*. Consistently, *in vivo* studies validated their robust, concentration-related pro-neurogenic and bone-regenerative effects, as demonstrated in a sciatic nerve injury model and a skull defect model. These findings underscore that the MOF6 scaffold possesses remarkable potential to simultaneously support nerve repair and bone regeneration, representing a novel approach to integrating neural and skeletal tissue engineering. Its dual functionality, excellent biocompatibility and tunable properties position it as a promising candidate for regenerative medicine applications, particularly for treating neuro-bone composite injuries in oral and maxillofacial surgery, trauma and other related fields. However, further validation in clinical-relevant models (e.g. alveolar socket defect combined with nerve injury) and elucidation of the underlying molecular mechanisms are required before translational application.

## Data Availability

The dataset used and/or analyzed during the current study are available from the corresponding author on reasonable request.
